# Comparison of Bacterial Communities in Sands and Water at Beaches with Bacterial Water Quality Violations

**DOI:** 10.1371/journal.pone.0090815

**Published:** 2014-03-05

**Authors:** Elizabeth Halliday, Sandra L. McLellan, Linda A. Amaral-Zettler, Mitchell L. Sogin, Rebecca J. Gast

**Affiliations:** 1 Biology Department, Woods Hole Oceanographic Institution, Woods Hole, Massachusetts, United States of America; 2 School of Freshwater Sciences, Great Lakes Water Institute, University of Wisconsin-Milwaukee, Milwaukee, Wisconsin, United States of America; 3 Josephine Bay Paul Center, Marine Biological Laboratory, Woods Hole, Massachusetts, United States of America; 4 Department of Geosciences, Brown University, Providence, Rhode Island, United States of America; National University of Singapore, Singapore

## Abstract

Recreational water quality, as measured by culturable fecal indicator bacteria (FIB), may be influenced by persistent populations of these bacteria in local sands or wrack, in addition to varied fecal inputs from human and/or animal sources. In this study, pyrosequencing was used to generate short sequence tags of the 16S hypervariable region ribosomal DNA from shallow water samples and from sand samples collected at the high tide line and at the intertidal water line at sites with and without FIB exceedance events. These data were used to examine the sand and water bacterial communities to assess the similarity between samples, and to determine the impact of water quality exceedance events on the community composition. Sequences belonging to a group of bacteria previously identified as alternative fecal indicators were also analyzed in relationship to water quality violation events. We found that sand and water samples hosted distinctly different overall bacterial communities, and there was greater similarity in the community composition between coastal water samples from two distant sites. The dissimilarity between high tide and intertidal sand bacterial communities, although more similar to each other than to water, corresponded to greater tidal range between the samples. Within the group of alternative fecal indicators greater similarity was observed within sand and water from the same site, likely reflecting the anthropogenic contribution at each beach. This study supports the growing evidence that community-based molecular tools can be leveraged to identify the sources and potential impact of fecal pollution in the environment, and furthermore suggests that a more diverse bacterial community in beach sand and water may reflect a less contaminated site and better water quality.

## Introduction

Fecal indicator bacteria are increasingly well-documented in sands at a range of freshwater and marine beaches of varied climates [1], [2], [3], [4], [5], bringing into question whether their ability to persist in the surfzone environment compromises their utility as proxies for the risk of contact with pathogens in recreational waters. Contaminated sands may impact beachgoers either by negatively contributing to bathing water quality through cycles of deposition and resuspension of bacteria between sand and water [6], [7], [8], or perhaps be more directly harmful through physical contact with sands or ingestion of sands. Epidemiological studies that examined the health outcomes associated with beach sand activities revealed that increased interaction with sands (e.g. digging in, being buried in) corresponded to increased outcomes of illness [9], most commonly gastrointestinal illness but also including skin, eye, ear and respiratory infections. Interaction with sands that have a higher amount of fecal pollution (as measured by both molecular and culture-based quantification of the marine fecal indicator *Enterococcus*) also corresponded to increased outcomes of illness among beachgoers [10].

To date, a variety of viral, bacterial and eukaryotic human pathogens have been recovered from environmental beach sands [11], [12], [13], [14], but it is difficult to predict which specific pathogens may be present at a beach at any given time due to variations in sources of human and animal fecal pollution, as well as whether the fecal pathogens detected in sands are abundant enough to present a significant risk of illness [15]. In the environment, it is likely that fecal indicators and pathogens respond differently to the complex interactions between environmental variables such as moisture, temperature and sunlight [11], [12], [16], [17], [18] as well as to the ecological pressures presented by the indigenous microbial community [19], [20]. While intertidal beach sands are frequently in contact with the overlying water, previous studies have indicated that biofilm formation on sand grains effectively maintains separation between bacterial communities in sands, porewater, and overlying water [21] and that sands and sediments have distinctly different communities than those found in overlying water [22], [23]. While recent studies have indicated that water quality and sand quality are linked [24], [25], we have a limited understanding about how the complex environmental bacterial communities in sands, which may include fecal indicators and pathogens, are related to the putative pollution events that are detected through routine culture-based water quality monitoring. We especially lack information about how episodic water quality violations affect the bacteriological quality of surfzone sands, and whether signatures of human contamination can be identified in the sand during water quality violation events or at other times, which could help us better understand how human contamination persists in the environment and impacts humans [26].

The goal of this study was to leverage pyrosequencing datasets of 16S hypervariable region ribosomal DNA to survey total and constituent components of the bacterial communities at two beaches. Samples of dry sand, intertidal sand, and the overlying waters were collected during two-week periods at locations on the west and east coast of the United States, on days when water quality violations occurred as well as on days representative of baseline fecal indicator bacteria concentrations. At one site (Avalon, CA) waters frequently violate water quality standards and have been identified as being impacted by decaying sewage infrastructure; at the second site (Provincetown, MA) water quality violations are infrequent and of unknown origin, but have a history of occurring during both wet and dry weather.

Short sequence tags were used to examine the total bacterial community composition, the presence of sequence tags belonging to the marine fecal indicator *Enterococcus*, and the presence of a broader group of alternative fecal indicators derived from studies of human sewage [27]. Pyrosequencing was particularly advantageous for this combination of broad and specific queries, since the depth of sampling enables detection of many low-abundance members of the community and can document shifts in the community structure over time [21], [28]. Molecular methods (e.g., ARISA, T-RFLP, clone libraries and 454 sequencing) have been used to document bacterial diversity and community structure in similar environments such as submerged marine sediments [29], [30], lake sediments [31], and shallow subtidal sands [21], [23], [32], [33], and these previous studies focused on the impact of temporal/spatial, physical, chemical or biological disturbance events on bacterial community structure. The results presented here expand the body of information on marine sand and water microbial communities by utilizing sequence tags to examine the impact of water quality exceedance events due to suspected anthropogenic input.

## Materials and Methods

### Field Sites and Sample Collection

Water, wet sand (covered in approximately 10 cm of water) and dry sand at the high tide line were sampled at Avalon Bay Beach (Catalina Island, CA, **Figure S1**) from the end of July through the beginning of September, 2007. The site locations and qPCR methods used for enumerating enterococci in sands are described in detail elsewhere [34]. Briefly, sand cores were collected in triplicate by hand in 50 mL sterile Falcon tubes from three sites spanning a 200 m along-shore transect. Water from the sites was filtered onto 47 mm 0.22 µm pore size Durapore® filters (GVWP04700; Millipore) and 100 mL was also filtered to enumerate culturable enterococci using the EPA 1600 method [35]. All samples for DNA analysis were frozen and shipped to Woods Hole, MA, where they were kept at −80°C prior to genomic DNA extraction. *Enterococcus* spp. were quantified via qPCR using primers targeting the 23S rDNA [36]. Wet and dry sand from three days, each a week apart, were chosen for pyrosequencing analysis based on differences in water quality as per official beach closures, and also based on the relative amount of enterococci DNA present in the sands. On August 11^th^ and August 18^th^ the water complied with bacterial health standards, but sands differed in the amount of enterococci as detected by qPCR with August 11^th^ having relatively elevated enterococci and August 18^th^ having a low level of enterococci [34]. The third time point, August 25^th^, corresponded to a violation of the bacterial water quality standard and elevated enterococci in sands. These samples were designated as Avalon (AV) water, wet sand or dry sand (H/W/D respectively) from day 1, 2, or 3 (detailed in **Table 1** **)**.

**Table 1:** Environmental samples sequenced in this study.

Avalon Bay, CA							
Archived Sample ID (VAMPS):	#Sequence Tags	Species Richness	Sample	Date:	Abb. in paper:	>104 CFU?	Culturable ENT density
RJG_PTW_Bv6v4_AVB_0001_2007_08_11	35993	686	Water	08-11-2007	AV 1H	No*	55+/−123 CFU/100 mL^1^
RJG_PTW_Bv6v4_AVB_0002_2007_08_18	21647	470	Water	08-18-2007	AV 2H	No*	156+/−176 CFU/100 mL^1^
RJG_PTW_Bv6v4_AVB_0003_2007_08_25	27480	506	Water	08-25-2007	AV 3H	Yes	147+/−42 CFU/100 mL^1^
RJG_BSC_Bv6_AV081107W	9269	363	Wet Sand	08-11-2007	AV 1W	No	256+/−1056 MPN/100 g sand^2^
RJG_BSC_Bv6_AV081807W	8050	342	Wet Sand	08-18-2007	AV 2W	No	124+/−317 MPN/100 g sand^2^
RJG_BSC_Bv6_AV082507W	9437	399	Wet Sand	08-25-2007	AV 3W	Yes	83+/−63 MPN/100 g sand^2^
RJG_BSC_Bv6_AV081107D	9477	391	Dry Sand	08-11-2007	AV 1D	No	N/A
RJG_BSC_Bv6_AV081807D	11006	388	Dry Sand	08-18-2007	AV 2D	No	N/A
RJG_BSC_Bv6_AV082507D	11907	445	Dry Sand	08-25-2007	AV 3D	Yes	N/A
**Provincetown, MA**							
**Archived Sample ID (VAMPS):**	**#Sequence Tags**	**Species Richness**	**Sample**	**Date:**	**Abb. in paper:**	**>104 CFU?**	**Culturable ENT density**
RJG_PTW_Bv6v4_PTW_0001_2009_06_29	22757	433	Water	06-29-2009	PTW 1H	Yes	140 CFU/100 mL^3^
RJG_PTW_Bv6v4_PTW_0002_2009_06_30	26481	592	Water	06-30-2009	PTW 2H	No	35 CFU/100 mL^3^
RJG_PTW_Bv6v4_PTW_0003_2009_07_07	27124	624	Water	07-05-2009	PTW 3H	No	30 CFU/100 mL^3^
RJG_PTW_Bv6v4_PTW_0004_2009_07_12	30829	677	Water	07-12-2009	PTW 4H	Yes	185 CFU/100 mL^3^
RJG_PTW_Bv6v4_PTW_0005_2009_07_13	30569	662	Water	07-13-2009	PTW 5H	No	90 CFU/100 mL^3^
RJG_PTW_Bv6v4_PTW_0006_2009_06_29	43267	604	Wet Sand	06-29-2009	PTW 1W	Yes	169 CFU/100 g dry weight sand^3^
RJG_PTW_Bv6v4_PTW_0007_2009_06_30	38079	645	Wet Sand	06-30-2009	PTW 2W	No	95 CFU/100 g dry weight sand^3^
RJG_PTW_Bv6v4_PTW_0008_2009_07_07	38282	648	Wet Sand	07-05-2009	PTW 3W	No	715 CFU/100 g sand^3^
RJG_PTW_Bv6v4_PTW_0009_2009_07_12	41572	668	Wet Sand	07-12-2009	PTW 4W	Yes	858 CFU/100 g sand^3^
RJG_PTW_Bv6v4_PTW_0010_2009_07_13	31806	514	Wet Sand	07-13-2009	PTW 5W	No	217 CFU/100 g sand^3^
RJG_PTW_Bv6v4_PTW_0011_2009_06_29	28326	619	Dry Sand	06-29-2009	PTW 1D	Yes	2927 CFU/100 g sand^3^
RJG_PTW_Bv6v4_PTW_0012_2009_06_30	36023	730	Dry Sand	06-30-2009	PTW 2D	No	2419 CFU/100 g sand^3^
RJG_PTW_Bv6v4_PTW_0013_2009_07_07	28202	600	Dry Sand	07-05-2009	PTW 3D	No	329 CFU/100 g sand^3^
RJG_PTW_Bv6v4_PTW_0014_2009_07_12	29688	605	Dry Sand	07-12-2009	PTW 4D	Yes	10745 CFU/100 g sand^3^
RJG_PTW_Bv6v4_PTW_0015_2009_07_13	33590	660	Dry Sand	07-13-2009	PTW 5D	No	342 CFU/100 g sand^3^

**Figure 1:**
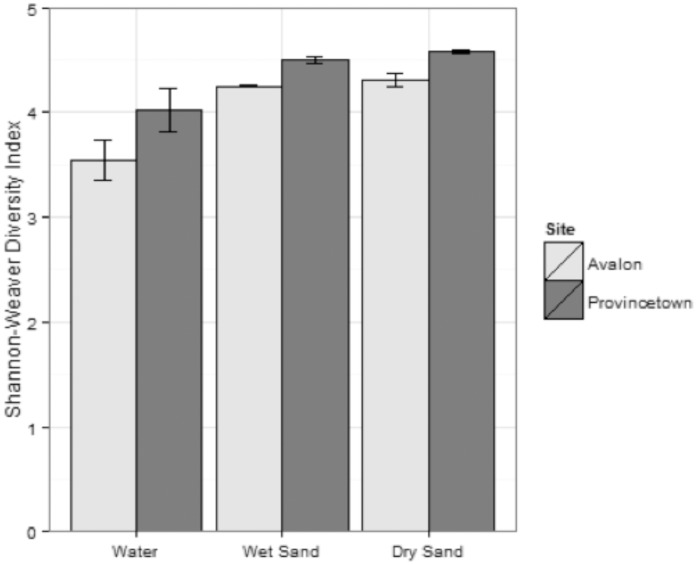
Diversity of bacterial communities in sands and water at Avalon and Provincetown. Error bars indicate two standard errors for the indices calculated for individual samples (n = 3 (days) at Avalon, n = 5 (days) at Provincetown).

1 Geometric mean +/− standard deviation of samples collected by SCCWRP at three sites along the beach (Figure 1) at 8am using USEPA1600.

*Based on posted results from health department, which utilized USEPA Method 1600 to detect CFU/100 mL. On the 25^th^, all water quality samples were >104 CFU/100 mL, whereas the other days were more variable as illustrated in the standard deviation of the SCCWRP results.

2 MPN/100 g sand, calculated by SCCWRP from a single sample collected on the beach by SCCWRP using the IDEXX Enterolert assay.

3 Calculated by E. Halliday using USEPA Method 1600 for water and modified for sands as previously described [51], normalizing CFU to dry weight sand.

In the summer of 2009, the beach at 333 Commercial St. (Provincetown, MA, **Figure S1**) was sampled three days per week from mid June through the end of July. Samples from five days that represented a range of water quality and wet weather conditions were chosen for pyrosequencing analysis. These samples were designated as Provincetown (PTW) water, wet sand or dry sand (H/W/D respectively) from days numbered 1–5 (detailed in **Table 1**). Ancillary environmental data were collected at each sampling event, including the temperature of the sample (water temperature, wet sand temperature, dry sand temperature), the tidal range prior to the sampling event (reflecting variations in spring and neap cycles), the level of the tide during the sampling event, the amount of precipitation within the previous 24 h, and the amount of enterococci cultured from the sample.

#### Ethics statement

Permits are not required to collect water and beach sand samples from the public beaches in California and Massachusetts. No protected species were sampled.

### Genomic DNA Extraction and 454 Pyrosequencing

The UltraClean Mega Prep soil DNA kit (MoBio Laboratories, Inc., Solana Beach, CA) was used to extract genomic DNA from 9.0 g of a wet-weight sand composite of three replicates taken from the sand surface at the beach. DNA was extracted from filtered water samples using a modified combination of hot detergent lysis buffer and mechanical disruption as previously described [37]. Eluted DNA was checked for purity with a NanoDrop spectrophotometer before PCR was used to amplify the V6 hypervariable region. Avalon sand samples were sequenced first, on a Roche Genome Sequencer GS-FLX using standard protocols [38], and at the time were limited toV6 amplicon libraries of tag sequences 60 bp long. By 2009 and the second sequencing run, the sequence tag read length had improved to 250 bp, so combined V4 and V6 amplicon libraries were sequenced. In all samples, sequences of adapters and primers were trimmed and low-quality reads removed as described previously [39]. Taxonomy was assigned through the Global Alignment for Sequence Taxonomy (GAST) using a 16S hypervariable region reference database [39]. GAST assigns taxonomy to a tag based on a two-thirds majority vote of the taxonomy of the nearest full-length relatives using a threshold of >80% sequence similarity. Taxonomical assignments within samples are archived and publicly available for comparison on the Visualization and Analysis of Microbial Population Structures (VAMPS) project website (http://www.vamps.mbl.edu).

### Data Analysis

To compare diversity among sample types and sites, 8050 sequence tags were randomly subsampled from each sample to minimize the impact of varied sequencing depth among samples. For all other analyses, sequence tag data was normalized to relative abundance within the sample for analysis and visualization. The statistical software package PRIMER-E [40] was used to analyze the relative abundance data of sequence tags successfully assigned to taxa within our samples, with the one-way ANOSIM testing significance of difference between groups of samples based on differences in site, sample type and water quality violation events. Multidimensional scaling analysis was used to generate graphical representations with Non-metric Multi-dimensional Scaling (NMDS) plots of relative differences in community composition between samples from Avalon and Provincetown. The BIOENV rank-correlation procedure was used with the Provincetown samples to determine which combinations of variables best explain patterns in the sequence tag abundance data. The SIMPER routine was used to identify the specific sequence tags with the greatest contribution to the dissimilarity observed between samples. To assess if fecal organisms present in beach sand were associated with sewage, sequence tags belonging to three orders (Bifidobacteriales, Bacteroidales and Clostridiales) were extracted from the total datasets for comparison between samples. These were directly compared to sewage datasets (SML_SWG_Bv6) [27] archived on the VAMPS website.

## Results

### Community Diversity and Structure

Thirty-nine phyla were represented among the 630,858 total and 2,349 unique bacterial sequence tags recovered from the twenty-four sand and water samples in this study. Sequencing depth and total species richness within individual samples are documented in **Table 1**, and normalized species richness for each sample is illustrated in the rarefaction curves of **Figure S2**. The Shannon-Weaver diversity index was calculated for each sample, and the average indices for water, wet sand, and dry sand at each of the sites (**Figure 1**) show bacterial communities in sand to be more diverse than those in water, and that greater diversity was present at the Provincetown site than at the Avalon site.

**Figure 2:**
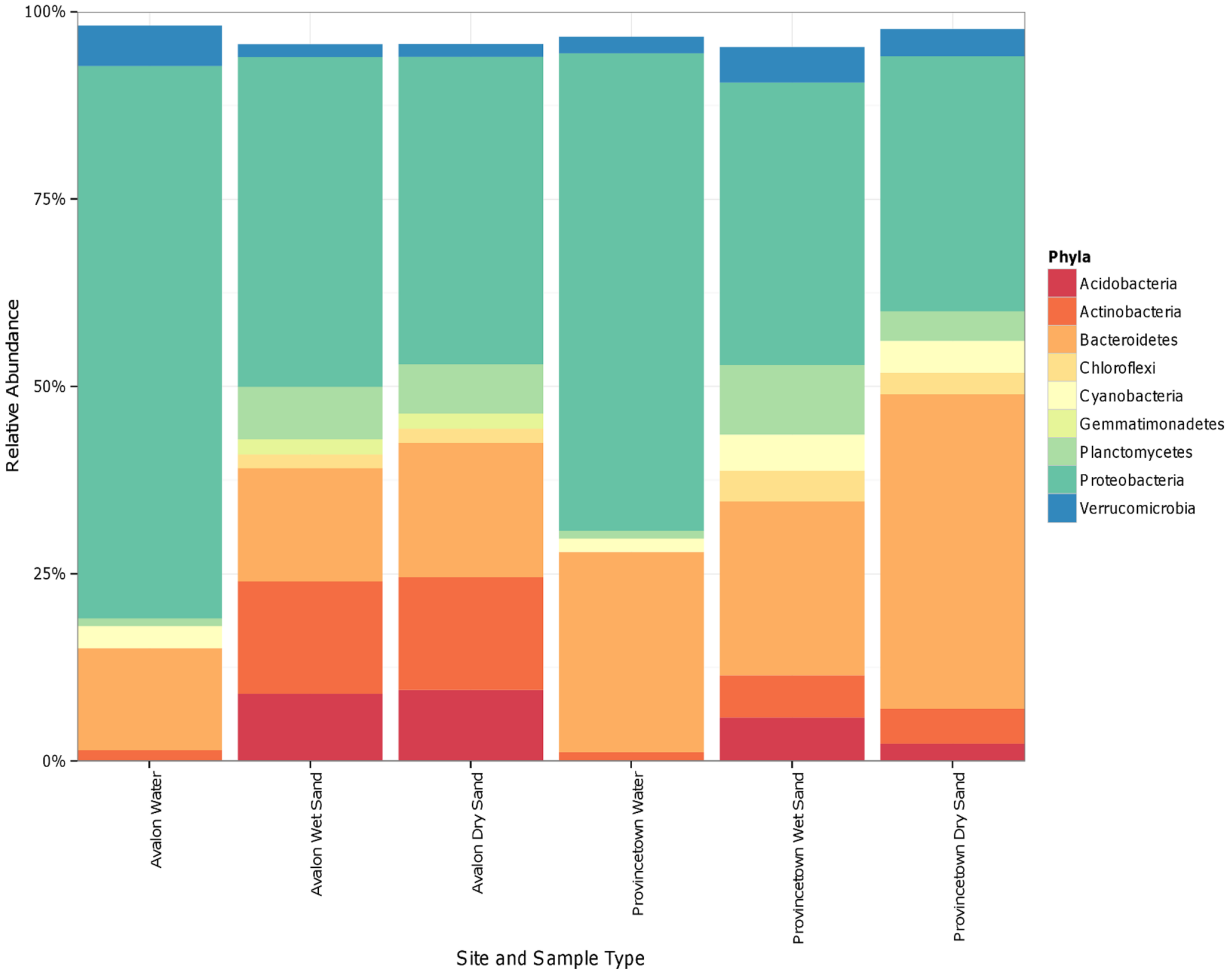
Relative abundance of phyla containing >1% of total sequence tags in water and sand samples.

The average distribution of sequence tags among dominant phyla in each sample type is presented in **Figure 2**. Proteobacteria dominate the sequence tags from water samples regardless of site; within this phylum, the orders Alphaproteobacteria and Gammaproteobacteria contain the majority of sequence tags, which are present in a ratio of approximately 2∶1 respectively. Sequence tags assigned to SAR11 (*Pelagibacter spp*.) were the most abundant unique tag sequence in water samples accounting for, on average, 15% of the sequence tags in Provincetown water and 25% of the sequence tags in Avalon water samples. Likewise, SAR116 and SAR 86 accounted for 2% and 5% of the total tags in Provincetown water samples, and 4% and 8% of total tags in Avalon water samples. In water samples, the phyla Bacteroidetes followed Proteobacteria in dominance, with strong representation from species in the *Flavobacteriaceae* family. Several species within the *Flavobacteriaceae* family had abundances >1% of the total tags per sample at both sites, and one unique sequence (unidentified to genus) represented 7% and 6% of tags at Avalon and Provincetown as well as 1% of tags in wet sands at both sites and dry sands at Provincetown. The Cyanobacteria were also abundant, including a unique *Synechococcus spp.* sequence that accounted for 2% of total tags in water at both sites. Proteobacteria, Bacteroidetes, Cyanobacteria and the Verrucomicrobia together included approximately 95% of the bacterial sequence tags from waters at Avalon and Provincetown.

In the Avalon beach sands, five phyla collectively contained >90% of the successfully identified tags (**Figure 2**); these were the Proteobacteria, Bacteroidetes, Actinobacteria, Planctomycetes and Acidobacteria. Three other phyla, the Chloroflexi, Gemmatimonadetes and Verrucomicrobia, were present at appreciable relative abundance (>1% of total phyla tags). Within the Avalon sand Proteobacteria, the Alphaproteobacteria and Gammaproteobacteria were again the most abundantly represented orders, but sequence tags from the Deltaproteobacteria were also relatively abundant, containing approximately 5% of the total sequence tags from sand samples. One unique sequence tag within the Gammaproteobacteria (belonging to the *Sinobacteraceae* family) accounted for 8% and 9% of tags in Avalon wet and dry sand samples, as well as 9% and 3% in Provincetown wet and dry sand samples. Provincetown sands were dominated by the same phyla found at Avalon, but phyla that were minor components at Avalon (Chloroflexi, Cyanobacteria, and Verrucomicrobia) claimed a greater proportion of sequence tags in Provincetown sands, generally at the expense of the Acidobacteria and Actinobacteria.

Among unique sequence tags with abundances accounting for >1% of the total sequence tags in sand types at each of the sites, several species were notably present in both Provincetown and Avalon sands. Within the Acidobacteria, these included a *Chloroacidobacterium* spp. sequence tag which accounted for 2% of tags in wet and dry sand at both sites, and a sequence assigned only to the class Holophage that accounted for 2% of tags in Provincetown wet sand samples and 6% of tags in Avalon wet and dry sand samples.

Within the Actinobacteria, a unique sequence tag belonging to the order Acidimicrobiales accounted for 9% of tags in Avalon wet and dry sand and 3% of tags in Provincetown wet sand. A unique sequence tag identified as *Iamia* spp. accounted for 3% of tags from Avalon wet and dry sand, and 2% and 1% of the sequence tags from Provincetown wet and dry sands respectively. *Nitriliruptor* spp. constituted 1% of sequence tags in Provincetown dry sand as well as in Avalon wet and dry sand.

Within the Bacteroidetes, the Sphingobacteriales were strongly represented in sands including several unique sequence tags that were abundant at all sites; these included a sequence identified as *Haliscomenobacter* that accounted for 3–4% of tags in all sand samples at both sites, a sequence identified to the family *Rhodothermaceae* that included 11% of tags from Provincetown dry sand, 3% of tags in Provincetown wet sand, 2% of Avalon wet tags and 3% of Avalon dry sand tags.

### Community Composition based on Shared Sequence Tags

**Figure 3:**
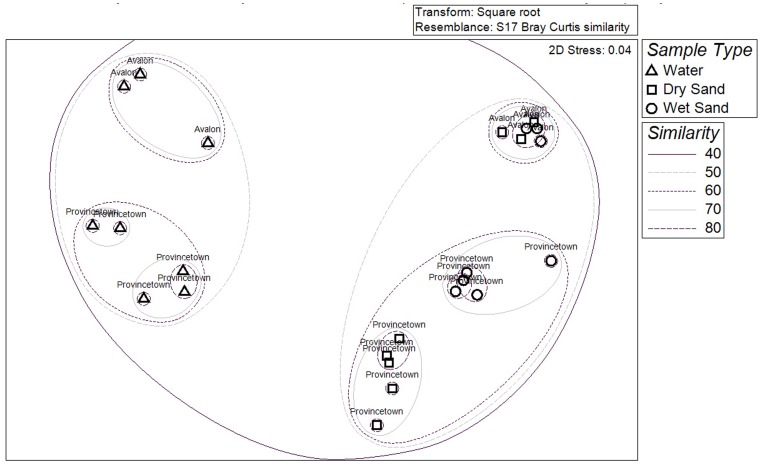
NMDS plot of total bacterial community composition in samples.

Although the bacterial phyla and some of the species dominating beach sand or water communities at both sites were broadly similar, analysis of the distribution of specific tag sequences among samples yielded a more localized view of community composition. A one-way Analysis of Similarity test (ANOSIM) rejected the null hypothesis that there was no significant difference in total community structure at the level of individual sequences based on the sample type (water, wet sand, dry sand) with a Global R of 0.698 (p≤0.001). Likewise, a one-way ANOSIM rejected the null hypothesis that there were no significant differences in total community structure at the level of individual sequences between sites (Avalon vs. Provincetown) with a weak but still significant Global R of 0.3 (p≤0.01). The NMDS ordination (**Figure 3**) illustrates the split between water samples and sand samples (groupings differentiated with >50% sequence tag similarity) and within that, samples are further separated by site (differentiated with >60% sequence tag similarity). Within the Provincetown sand samples, there are further groupings that correspond to wet and dry sand, distinguishing communities that are geographically separated by a tidal range that is greater than Avalon’s. Although other temporal influences cannot be precluded, the differences between water samples at Provincetown may in part be attributed to tidal stage, as water samples collected during high (1H and 2H) and low tides (3H, 4H, 5H) are still distinct groups despite being quite similar to each other (<70%).

### Environmental Variables Influencing Total Community Composition at Provincetown

Environmental variables including sample temperature, tidal range, water level, precipitation and amount of enterococci were used in the BIOENV rank-correlation procedure, in which variables or combinations of variables are selected based on how they best explain patterns in the sequence tag abundance data. Among Provincetown water samples, the variables water temperature and tidal range were selected together to produce the best possible r = 0.758. The only relationship between the environmental variables and wet sand bacterial samples was the single selection of tidal range with an r = 0.164. Dry sand samples had a better relationship to environmental variables with r = 0.467 for dry sand temperature. Thus, sample temperature and tidal range emerged as the most important environmental variables shaping total community structure in water, and sand community structure was explained to a lesser extent by these variables.

### Community Composition of Alternative Indicator Sequence tag Sample Subsets

A previous study used 454 sequencing technology to examine both human waste and wastewater treatment plant influent, and identified a group of bacteria as potential alternative indicators of fecal pollution belonging to the orders Clostridiales, Bifidobacteriales and Bacteroidales [27]. In our study, sequence tags identified within these orders from the total beach sand and water tag datasets were compared to the sewage tag datasets to determine whether there were trends in any of the sample types that corresponded to water quality violation events. Overall, although all samples contained sequence tags belonging to these orders, there was very little overlap between the specific sequence tags recovered from sewage and environmental samples. The only putative tag signature shared between sewage and environmental samples belonged to the order Clostridiales and was identified as *Roseburia.* This sequence tag was found in Provincetown water and wet sand on the day of and following the dry weather exceedance, and in water and dry sand the day of and following the wet-weather exceedance. This tag was also present in Avalon water samples on the days of elevated enterococci and dry weather exceedance events. Consistent with its hypothesized role as a sewage indicator, the relative abundance of the *Roseburia* tag was two to three orders of magnitude lower in environmental samples than would typically be found in sewage.

The one-way ANOSIM showed that there were no significant differences in the distribution of Clostridiales, Bifidobacteriales and Bacteroidales when samples were classified as having acceptable or unacceptable water quality (outlined in **Table 1**). However, ANOSIM showed significant differences among these orders when samples were grouped by site (R = 0.545, p = 0.001) or sample type (R = 0.298, p = 0.001). The SIMPER routine revealed that the differences between Clostridiales, Bifidobacteriales and Bacteroidales among sample types were primarily driven by the dominance of specific sewage sequence tags from the Bacteroidales *(Bacteroides, Parabacteroides, Paludibacter*) and Clostridiales (*Blauthia* and *Fecalibacterium)*, all of which were much more abundant in sewage than in sands or waters. Sewage samples from the studies of McLellan et al. [27] were found to be far less heterogeneous than the environmental samples collected in this study, as SIMPER revealed that the within-group similarity of alternative indicators in the sewage community was 77%, compared to 46% similarity within the water samples, and only 32% and 35% similarity for the wet sand and dry sand samples. Alternative fecal indicators differentiating Avalon samples were primarily sequence tags identified to the family *Ruminococcaceae* (genus unresolved), and the genus *Alistipes* within the Bacteroidales. Provincetown samples were differentiated by a relative abundance of tags belonging to Clostridiales (*Robinsonella*, *Fusibacter* and *Acetivibrio*).

### Environmental Variables Influence Alternative Fecal Indicators at Provincetown

When the BIOENV procedure was run with the same environmental variables but with the smaller subset of tags in the orders Clostridiales, Bifidobacteriales and Bacteroidales rather than the entire data set, the correlations significantly improved. The variable water temperature was found to best explain the patterns of abundance within the group of potentially sewage-associated orders recovered from water samples (r = 0.867), and dry sand temperature best explained the patterns of abundance within the group of potentially sewage-associated orders recovered from dry sands (r = 0.837), suggesting that this subset of the total community is more strongly influenced by temperature (or perhaps that bacterial loads to the beach covary with temperature) than by traditional fecal indicator abundance.

All of the water samples collected during exceedance events at both sites contained tags assigned to the genus *Enterococcus.* Some of the *Enterococcus* tags from Avalon also had a species level assignation *(E. ratii and E. colombae)* corresponding to GenBank sequence acquisitions from studies of unhealthy rats and pigeons, respectively. Although sands were often enriched in culturable *Enterococcus* compared to water (**Table 1**), only a single sand sample (Avalon dry sand, day of exceedance) contained *Enterococcus* sequence tags.

## Discussion

Intertidal sands and the overlying water proved to have distinct bacterial communities, with greater similarity observed between coastal water samples from two distant sites than between the water and sand from the same site. The differentiation between Provincetown wet and dry sand, a phenomenon not observed among Avalon sands, likely reflects the greater tidal range at Provincetown (2–4 m) and thus stronger physical separation and more distinct environmental conditions between the intertidal and upper beach sand bacterial populations. Two recent studies also report observing similar differences in bacterial community structure between dry and wet sand [23], [25]. Temperature and daily tidal range appeared to explain some of the variation in community structure in water and dry sand samples at the beach in our study (Provincetown), suggesting that the dry sand community may develop differently from that of the wet sand due to lack of tidal wetting. Furthermore, although the species richness in samples from both sites was similar the differences in community composition between samples collected at Avalon versus those collected at Provincetown may suggest greater ecological health and resiliency to contamination at Provincetown, where bacterial exceedance events occur less frequently and water quality is generally quite good. Additional work comparing beaches with different contamination levels is needed to confirm this observation.

The majority of beach sand sequence tags from both locations belonged to the Acidobacteria, Actinobacteria, Bacteroidetes, Proteobacteria and the Planctomycetes. These are broadly similar to soil communities, as >90% of sequence tags from soils collected around the world have been have been classified within the Actinobacteria, Acidobacteria, Proteobacteria, Bacteroidetes and Firmicutes, with the relative abundance of these groups within samples strongly influenced by soil pH [41]. In particular, beach sand communities are broadly differentiated from both coastal seawater and soil by the relative abundance of the Planctomycetes, which at both sites are relatively more abundant in wet sand than dry sand. The enrichment of the Planctomycetes in wet beach sands may reflect this phyla’s frequent affiliation with organic detritus in the marine environment or participation in chitin degredation [42]. Coastal seawater samples at both sites in this study were dominated by tag sequences (such as *Pelagibacter*) that were not abundant in sand samples; this differs from the results of Cui et al. [23] who found subtropical water and intertidal sand shared relatively high abundances of a sequence tag identified as *Pseudoalteromonas*. This tag was present, but not dominant, in our samples; the sum of all unique sequences identified as *Pseudoalteromonas* species ranged from 0.03% to 0.7% of the total sample in both waters and sands. The same study [23] identified four species (*Nitriliruptor*, *Acidobacterium*, *Pseudomonas* and *Paracoccus*) uniquely abundant within subtropical backshore sand samples. These four species were recovered from the sand samples in this study, although they were taken from the upper and lower limits of the intertidal zone and not the “backshore” per se. A unique tag identified as *Nitrilirupter* accounted for 1% of tags in Avalon wet and dry sand as well as 1% of tags in Provincetown dry sand. A variety of *Paracoccus* tags were recovered from water and sand samples at all sites, with the highest relative abundance found in Provincetown dry sands (0.04%). Likewise a variety of *Pseudomonas* tags were recovered from all samples, all at similarly low relative abundances (approximately 0.01%). *Acidobacterium* was found at low relative abundance in Avalon wet sand (0.007%). However, the differentiations between Provincetown wet and dry sand communities was consistent with previous observations of the development of unique bacterial communities in different zones of beach sands [23], [25].

In contrast to total community, as a group the alternative fecal indicators were more similar among the sand and water collected at the same site, supporting the theory that while each of these beaches may be anthropogenically impacted, there are possibly regional differences in sewage/source profiles. Furthermore, the complexity of the alternative indicators in environmental samples compared to sewage makes it difficult to interpret the very minor overlap of a few specific sequence tags and may be suggestive of diffuse nonpoint source pollution. Our result is similar to the work by Shanks et al [43] where they determined that fecal associated OTUs were consistent nationwide across sewage samples, but the sewage OTUs identified as infrastructure associated varied among cities with a strong north/south latitudinal separation.

Although massively-parallel pyrosequencing tags of rDNA hypervariable regions provide unprecedented depth of sampling within the bacterial community, the short length of the sequence tags precludes identification of the majority of sequence tags to genera. It simply cannot reliably differentiate between strains, which is often the level of identification required to determine human health risk among a species of bacteria. Sequence tags from the indicator *Enterococcus* were recovered on days that had exceeded water quality standards, but the rarity of *Enterococcus* in this and other pyrosequencing datasets [27], [44] illustrates how the concentrations of indicators that cause concern from a monitoring perspective are relatively rare within the total community and not predictive of fecal bacteria in general (at least, based on this group of alternative indicators).

This study adds to the growing evidence that the community-based molecular tools used by microbial ecologists to study spatial and temporal variation and environmental disturbance events can be leveraged to study the sources and potential human health risks of fecal microbial pollution in the environment. In other studies, 16S-based pyrosequencing approaches have been used to broadly survey potential risks within sewage sludge and biosolids [45] and wastewater treatment plant samples [46], and with time these kinds of analyses can be completed with a broader range of potential source material. In terms of sourcing fecal pollution from animals or humans in surface waters, several community-based approaches have been developed that are analogous to current library-based microbial source tracking of single indicators [47]. For example, similarities of T-RFLP profiles of a coastal creek and potential human and animal fecal sources have been used to identify fecal sources and the extent to which contamination upstream impacts sites downstream [48]. In a case involving a limited number of environmental and local source samples, pyrosequencing of 16S rDNA derived from human and animal feces was used to examine the overlap between fecal sources and surface water communities, thereby discriminating which were likely dominant source material to the river [49]. Various approaches have also been used to identify constituent groups of human-specific fecal bacteria in environmental samples, including the pyrosequencing approach that was the basis for the group of alternative fecal indicators we analyzed in this study [27], as well as alternative approaches such as amplification of the V3 region of 16S rDNA combined with capillary-electrophoresis single strand conformation polymorphism (C-ESSCP) to fingerprint human feces and sewage effluents and identify dominant, human-specific bacteria [50]. However, previous studies have not considered the impact of putative sewage contamination events on the microbial community fingerprint of sand, sediment, or wrack. Here we have begun to assess whether such events can be identified in the dynamic marine beach environment by fecal signature tags. Further application of community-based methods to a wide array of environmental samples, sources and reservoirs may ultimately contribute to the diagnoses of bacterial pollution from unknown sources at beaches and in surface waters.

## Supporting Information

Figure S1 **Sites (indicated by black point) sampled at Avalon Bay, CA (left) and Provincetown Harbor, MA (right).** Samples were taken from the left and right of the Green Pleasure Pier in Avalon Bay and composited for this study, as described in the methods. Samples were taken from the left of MacMillan wharf in Provincetown Harbor, corresponding to the street address of 333 Commercial St. in Provincetown, MA. Both maps depict 7.5-minute series from the USGS National Map Viewer (http://viewer.nationalmap.gov/viewer).
(DOCX)

Figure S2 **Rarefaction Curves.** The number of unique species among 8050 randomly subsampled sequence tags from each environmental sample. See Table 1 for sample details.
(DOCX)
