# Supplementary material for: Comparison of Bacterial Communities in Sands and Water at Beaches with Bacterial Water Quality Violations
Source: PLoS One. 2014 Mar 5;9(3):e90815. doi: 10.1371/journal.pone.0090815 (PMC3944938; doi:10.1371/journal.pone.0090815)

**Supplementary Information**

**Figure S1: Sites (indicated by black point) sampled at Avalon Bay, CA (left) and Provincetown Harbor, MA (right).** Samples were taken from the left and right of the Green Pleasure Pier in Avalon Bay and composited for this study, as described in the methods. Samples were taken from the left of MacMillan wharf in Provincetown Harbor, corresponding to the street address of 333 Commercial St. in Provincetown, MA. Both maps depict 7.5-minute series from the USGS National Map Viewer (<http://viewer.nationalmap.gov/viewer>).


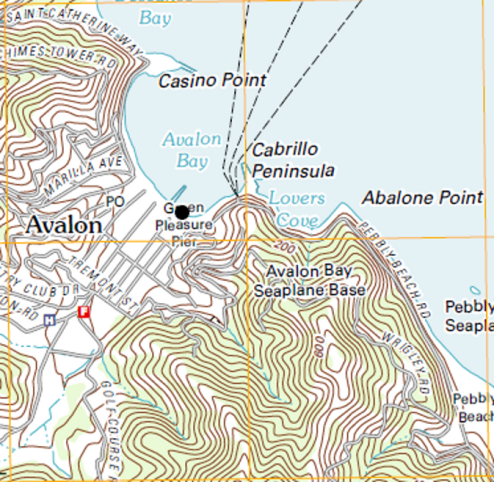

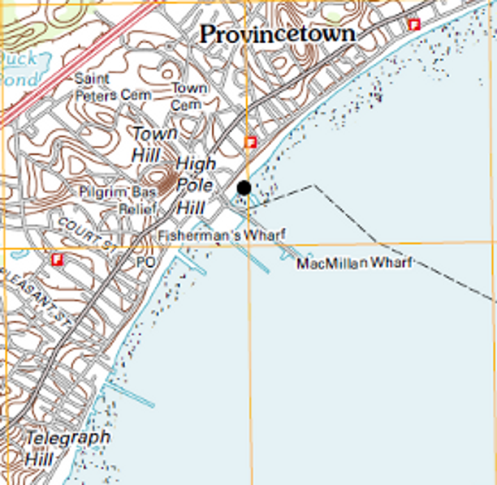

Supplement: Figure S1 — Sites (indicated by black point) sampled at Avalon Bay, CA (left) and Provincetown Harbor, MA (right). Samples were taken from the left and right of the Green Pleasure Pier in Avalon Bay and composited for this study, as described in the methods. Samples were taken from the left of MacMillan wharf in Provincetown Harbor, corresponding to the street address of 333 Commercial St. in Provincetown, MA. Both maps depict 7.5-minute series from the USGS National Map Viewer (http://viewer.nationalmap.gov/viewer). (DOCX) [file pone.0090815.s001.docx]
