# Supplementary material for: Comparison of Bacterial Communities in Sands and Water at Beaches with Bacterial Water Quality Violations
Source: PLoS One. 2014 Mar 5;9(3):e90815. doi: 10.1371/journal.pone.0090815 (PMC3944938; doi:10.1371/journal.pone.0090815)
Supplement: Figure S2 — Rarefaction Curves. The number of unique species among 8050 randomly subsampled sequence tags from each environmental sample. See Table 1 for sample details. (DOCX) [file pone.0090815.s002.docx]

**Figure S2: Rarefaction Curves.** The number of unique species among 8050 randomly subsampled sequence tags from each environmental sample. See Table 1 for sample details.
